# Three‐dimensional characterization of the active volumes of PTW microDiamond, microSilicon, and Diode E dosimetry detectors using a proton microbeam

**DOI:** 10.1002/mp.13705

**Published:** 2019-07-22

**Authors:** Daniela Poppinga, Rafael Kranzer, Ann‐Britt Ulrichs, Björn Delfs, Ulrich Giesen, Frank Langner, Björn Poppe, Hui Khee Looe

**Affiliations:** ^1^ PTW Freiburg Freiburg Germany; ^2^ University Clinic for Medical Radiation Physics Medical Campus Pius Hospital, Carl von Ossietzky University Oldenburg Germany; ^3^ Physikalisch‐Technische Bundesanstalt (PTB) Braunschweig Germany

**Keywords:** active detector volume, dosimetry, microSilicon, proton microbeam, response map

## Abstract

**Purpose:**

The purpose of this work is the three‐dimensional characterization of the active volumes of commercial solid‐state dosimetry detectors. Detailed knowledge of the dimensions of the detector’s active volume as well as the detector housing is of particular interest for small‐field photon dosimetry. As shown in previous publications from different groups, the design of the detector housing influences the detector signal for small photon fields. Therefore, detailed knowledge of the active volume dimension and the surrounding materials form the basis for accurate Monte Carlo simulations of the detector.

**Methods:**

A 10 MeV proton beam focused by the microbeam system of the Physikalisch‐Technische Bundesanstalt was used to measure two‐dimensional response maps of a synthetic diamond detector (microDiamond, type 60019, PTW Freiburg) and two silicon detectors (microSilicon, type 60023, PTW Freiburg and Diode E, type 60017, PTW Freiburg). In addition, the thickness of the active volume of the new microSilicon was measured using the method developed in a previous study.

**Results:**

The analysis of the response maps leads to active area of 1.18 mm^2^ for the Diode E, 1.75 mm^2^ for the microSilicon, and 3.91 mm^2^ for the microDiamond detector. The thickness of the active volume of the microSilicon detector was determined to be (17.8 ± 2) µm.

**Conclusions:**

This study provides detailed geometrical data of the dosimetric active volume of three different solid‐state detector types.

## 1 Introduction

In our previous work,^1^ we have established the use of a highly focused proton radiation provided by the microbeam system of the Physikalisch‐Technische Bundesanstalt (PTB) for the characterization of detector’s active volume. The diameters and thicknesses of the active volumes of several commercial solid‐state detectors for dosimetry in radiation therapy have been determined. As shown in the literature, the main perturbations of detector’s dose response at small photon beams are caused by the volume‐averaging effect due to the finite dimensions of the sensitive volume and the density of the detector components.^2^, ^3^, ^4^, ^5^, ^6^ Therefore, the measured signal of these detectors must be corrected using the detector‐dependent small‐field output correction factors as recommended in the recent IAEA report TRS 483.^7^ These correction factors were collected from the literature and were either determined experimentally, using Monte‐Carlo methods or a combination of both. Particularly for the determination of correction factors based on Monte Carlo simulations,^8^, ^9^, ^10^ the dimension of the active volume must be precisely known in order to enable correct modeling of the detector’s design.

Based on a previous work, the aim of this study is to extend the proton microbeam technique to obtain two‐dimensional response maps of radiation detectors and to allow the complete characterization of the active volumes. Two‐dimensional response maps of solid‐state detectors have been published by Butler et al.^11^, ^12^ and Marinelli et al.^13^ These studies used kilovoltage synchrotron radiation^11^, ^12^ and 35 kV x rays^13^ with beam diameters in the range of 100 µm^11^ or 200 µm.^13^ With the technique introduced in this work, the resolution of such measurements can be increased by a large factor by using a proton microbeam with a diameter in the range from about 10 µm at the detector surface and increasing to about 70 µm, depending on penetrated material. In addition, the same microbeam can be utilized to determine the thickness of the active detector volume; hence, the proton microbeam technique allows a complete three‐dimensional characterization of the detector’s active volume.

The active volumes of three detectors are characterized in this study: the synthetic diamond detector microDiamond (type 60019, PTW Freiburg), the unshielded silicon diode detector Diode E (type 60017, PTW Freiburg), and its successor the new unshielded silicon diode microSilicon (type 60023, PTW Freiburg). The thickness of the two former detectors has been determined in a previous study. Using the same methodology, the thickness of the new microSilicon detector will be evaluated. The motivation for the new silicon detector is an optimization of the basic dosimetric properties compared to its predecessor such as higher dose stability, lower dose per pulse dependence, and smaller sensitivity to temperature variation as well as an optimization especially for small photon fields. For this purpose, the entrance window was rendered more water equivalent by reducing the density of the casting compound on top of the silicon chip compared to the predecessor. As previous studies^3^, ^14^ demonstrated, the design of the detector housing influences the detector signal for small photon fields. Therefore, detailed knowledge of the active volume dimension and the surrounding materials form the basis for accurate Monte Carlo simulations of the detector. This basis is given within the scope of this study. This is especially important at this early stage, where no correction factors for this new detector are available yet. The results from this study will ensure uniformity among Monte Carlo studies on field output correction factors.

## 2 Materials and Methods

For all measurements in this study, a 10 MeV proton beam produced by a cyclotron accelerator of the PTB was used. The proton beam was focused to a diameter of approximately 10 µm using the microbeam facility. The proton rate was monitored using a monitoring system placed inside the exit nozzle of the microbeam system. The monitor system is based on a 40 µm scintillator foil backed with a 3 µm thick aluminum foil and a 5 µm Mylar foil, with the latter serving as a vacuum window.

### 2.A. Detectors

In this study, one sample of the synthetic diamond detector microDiamond (PTW type 60019), one sample of the silicon diode detector Diode E (PTW type 60017), and two samples of the silicon diode detector microSilicon (PTW type 60023) were investigated. All detectors were used with 0 V bias voltage and were read out by an Unidos E electrometer (PTW Freiburg,) with 1 fA resolution.

### 2.B. Two‐dimensional response maps

Two‐dimensional response maps were measured by positioning the detectors in air axially, that is, with the detector’s axis parallel to the beam, on a computer‐driven xy‐stage (Märzhäuser, Wetzlar, Germany) with a positioning uncertainty of ± 3 µm. The detectors were located about 1 mm below the exit nozzle of the beamline and the outer detector capsule was removed to minimize the material above the active volume. An automated routine was written based on visual C++ to synchronize the detector position, monitor signal, and electrometer signal during the measurements. For each data point, the detector signal was integrated over 2 seconds with a proton rate of approximately 3000 protons/s. Due to the limited beam time available for these measurements, the distance between two measurement points was set to 100 µm for the microDiamond detector and 50 µm for the silicon diodes.

Based on the measured two‐dimensional response maps, the active area *A* was determined by summing up the area of pixels with signal more than 50 % of the maximum. Assuming the active area is circular, the diameter *d* of the active area is given by

$$d=2\cdot \sqrt{\frac{A}{\pi }}$$

### 2.C. Determination of the active volume’s thickness

The thickness *t* of the active volume of the microSilicon detector was measured using the method developed in the previous study,^1^ based on the following equation:

$$t=\frac{I\cdot W}{n\cdot S\cdot e}$$

where *I* represents the detector current, *W* the energy expenditure per electron released in the silicon material (W = (3.6 ± 0.1) eV), *n* the proton rate, *S* the energy‐dependent proton stopping power in the active detector volume, and *e* represents the elementary charge.

The detector was positioned axially in front of the exit nozzle without the detector capsule. The proton beam was aligned to the center of the detector chip. The detector current *I* was measured three times over 60 s, while the proton rate *n* was monitored. As in the previous study, the mean proton energy at the active volume was determined by Monte Carlo simulations whereas the detector model, especially the composition and size of the casting compound above the silicon chip, was verified using a separate measurement. For this purpose, an aluminum absorber was placed between exit nozzle and detector surface and its thickness was varied. This was realized by combining a set of aluminum foils of 8 µm, 30 µm, and 50 µm thickness resulting in absorber thickness between 0 µm and 400 µm aluminum.

The Monte‐Carlo simulations were carried out using the FLUKA package version 2011.2x.3^15^ and the detector was modeled using the flair package according to the manufacturer blueprints with a casting material thickness of 0.48 mm and a material density of 1.16 g/cm^2^. The same set of parameters as in the previous study was used.

## 3 Results

### 3.A. Two‐dimensional response maps

**Figure 1:**
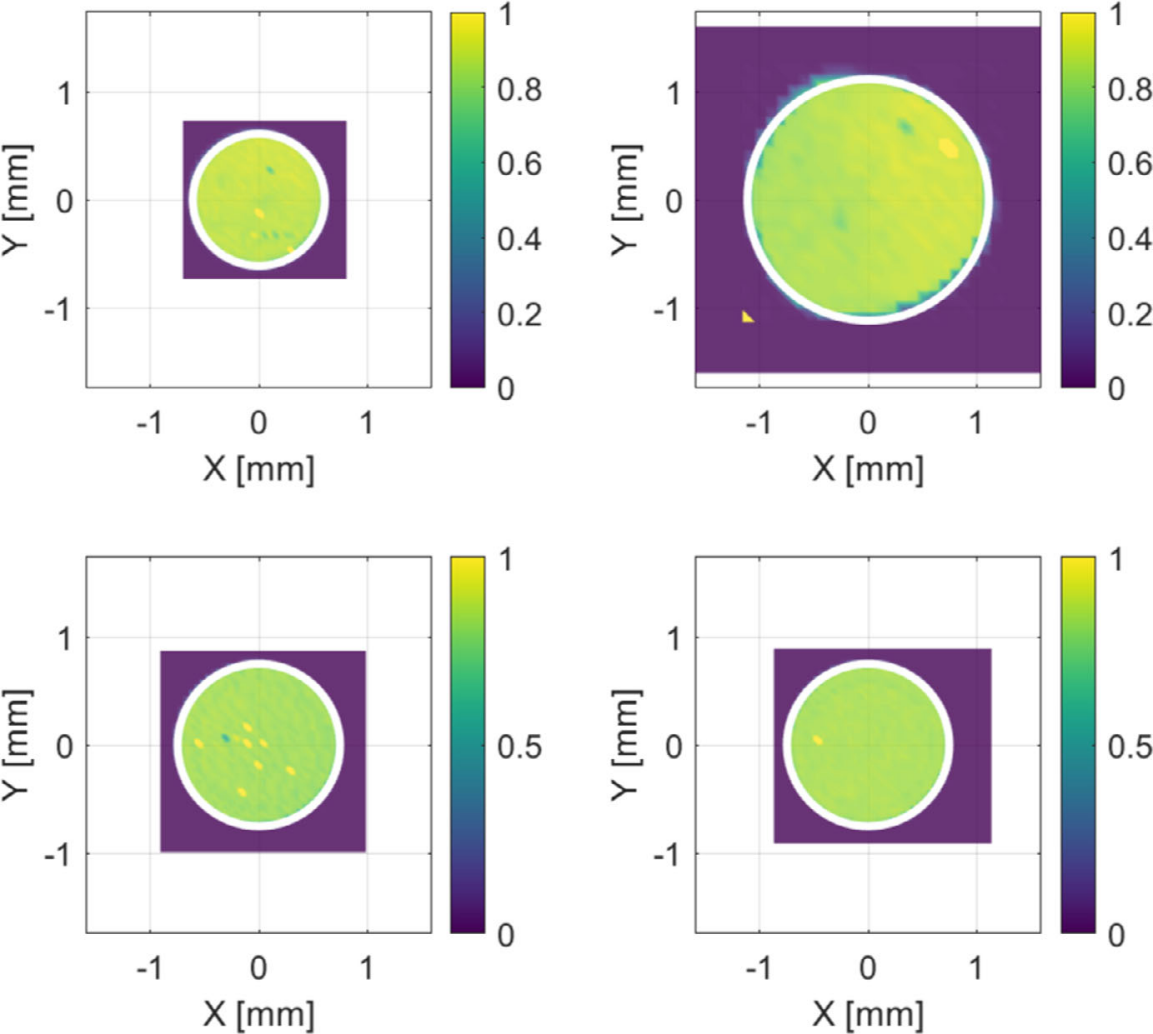
Two‐dimensional response maps. First row left: Diode E 60017, first row right: microDiamond 60019, second row: microSilicon 60023. The color scale represents the detector current normalized to the monitor signal. The white circle represents the calculated area of the active volume according to Table I. All data are displayed with same axis scaling.

The measured two‐dimensional response maps are shown in Fig. 1. For the Diode E 60017 detector, an active area of (1.18 ± 0.10) mm^2^ was measured, which corresponds to a diameter of (1.22 ± 0.05) mm. The active areas of the two samples of microSilicon detector were measured to be (1.75 ± 0.11) mm^2^ and (1.76 ± 0.11) mm^2^. These correspond to diameters of (1.49 ± 0.05) mm and (1.50 ± 0.05) mm, respectively. The measured active area of the microDiamond detector is (3.91 ± 0.25) mm^2^ corresponding to a diameter of (2.23 ± 0.10) mm. All results are summarized in Table 1. The calculated diameters are symbolized by the white circle in the Fig. 1.

**Table 1:** Measured area (*A*) and diameter (*d*) of the active volume and calculated thickness (*t*) of the active volume.

	*A* [mm^2^]	*d* [mm]	*t* [µm]
microDiamond 60019	3.91 (0.25)	2.23 (0.10)	2.0 (0.2)^1^
Diode E 60017	1.18 (0.10)	1.22 (0.05)	27.9 (3.2)^1^
microSilicon 60023 — I	1.75 (0.11)	1.49 (0.05)	17.7 (1.9)
microSilicon 60023 — II	1.76 (0.11)	1.50 (0.05)	17.9 (1.9)

The thickness of the microDiamond detector and Diode E 60017 was obtained from a previous study.^1^

Hot spots can be observed in the two‐dimensional response maps in Fig. 1, which are mainly caused by irregular count rates and disturbances of the monitor system.

### 3.B. Determination of the active volume’s thickness

**Figure 2:**
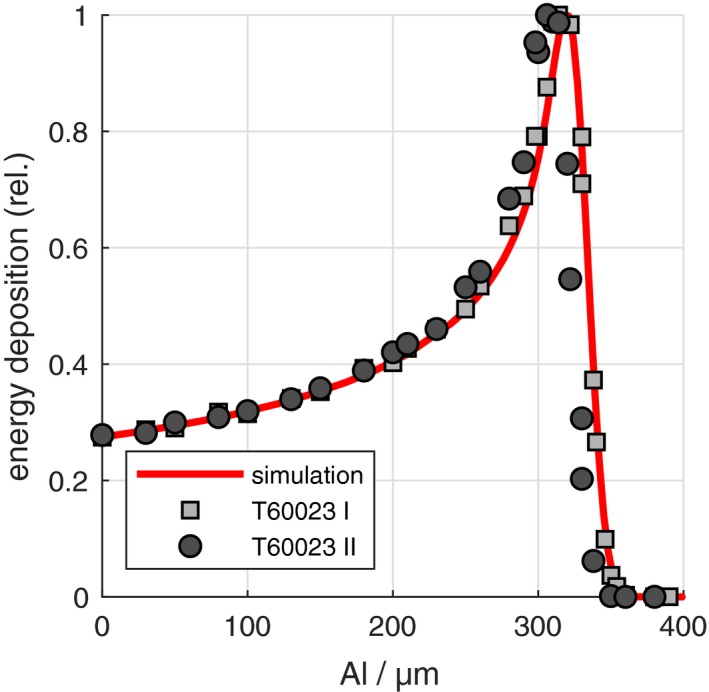
The data points represent the measured detector signal for different absorber thickness values. The red line represents the Monte Carlo simulation for the microSilicon detector T60023. [Color figure can be viewed at http://www.wileyonlinelibrary.com]

Figure 2 shows the normalized detector signal as a function of the absorber thickness. As shown in the figure, both microSilicon detectors show comparable results with a maximum signal corresponding to 310 µm aluminum absorber thickness, which agree to the Monte Carlo simulated results with the detectors modeled according to the manufactures’ blueprint (red line).

**Figure 3:**
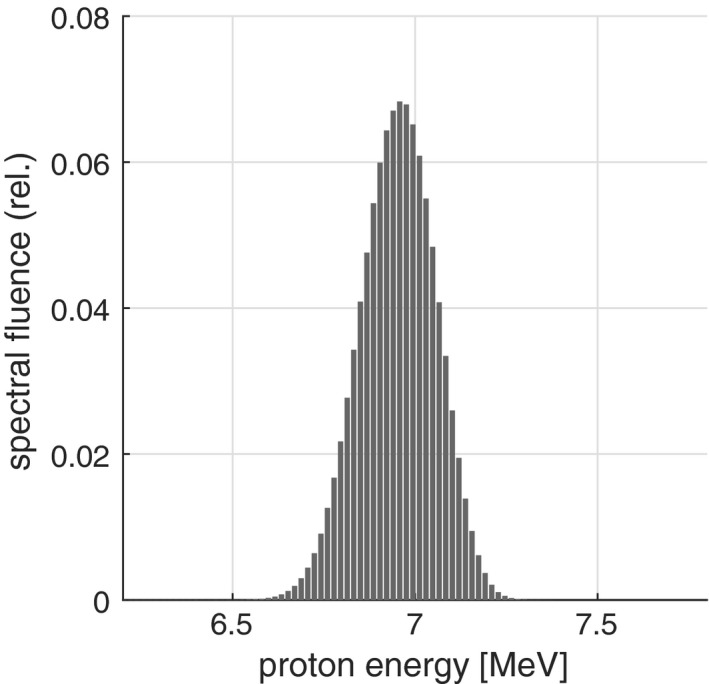
Monte‐Carlo calculated the spectral distribution of the proton beam at the surface of the detector’s active volume.

**Figure 4:**
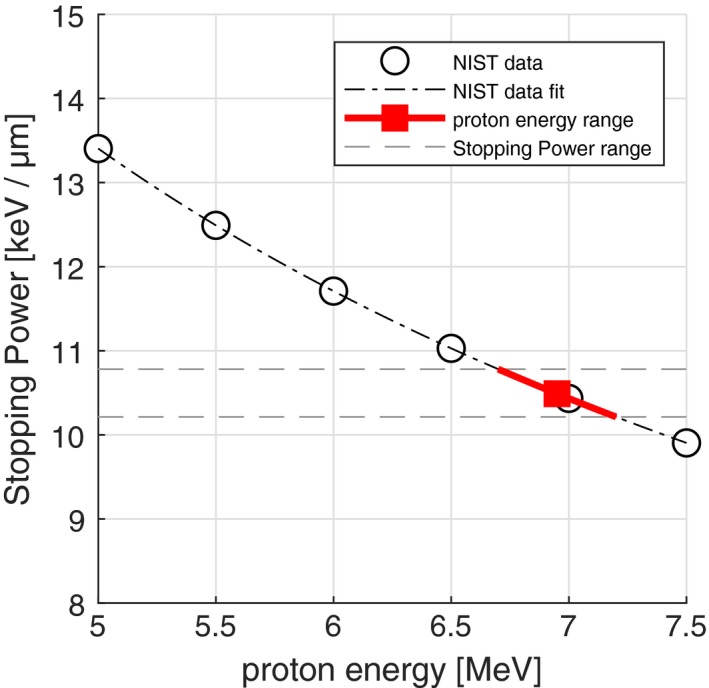
The stopping power of the proton beam in the detector’s active volume calculated based on the mean proton energy. The red square indicates the mean energy and the red line indicates the energy range of the beam (k = 1). [Color figure can be viewed at http://www.wileyonlinelibrary.com]

The agreement between simulations and measurements in Fig. 2 confirms the thickness and density of the casting material in front of the silicon chip. Moreover, this agreement confirms that the proton energy at the silicon chip can be calculated from the Monte Carlo simulation as shown in Fig. 3 resulting in a mean energy of 6.95 MeV without any additional absorber. The total proton stopping power at this mean energy was calculated based on the National Institute of Standards and Technology (NIST) PSTAR database.^16^ As shown in Fig. 4, the NIST values were fitted and the proton stopping power corresponding to the proton mean energy of 6.95 MeV was calculated to be (10.5 ± 0.3) keV µm^−1^.

According to equation 1, the thickness of the active volume can be determined using the calculated stopping power value, the measured detector signal without aluminum absorber, and the measured proton rate. The raw data are listed in Table 2. The thickness of the active volume of the two detector samples is calculated to be (17.7 ± 1.9) µm and (17.9 ± 1.9) µm.

**Table 2:** Measurement data to determine the thickness of the active volumes of the two microSilicon detector samples.

	S [keV µm^−1^]	I [pA]	n [s^−1^]	t [µm]
60023 — I	10.5 (0.3)	16.77 (0.01)	2037 (204)	17.7 (1.9)
		16.50 (0.01)	2007 (201)	17.7 (1.9)
		16.54 (0.01)	2008 (201)	17.7 (1.9)
60023 — II	10.5 (0.3)	16.67 (0.01)	2008 (201)	17.8 (1.9)
		16.61 (0.01)	1991 (199)	17.9 (1.9)
		16.93 (0.01)	2037 (204)	17.9 (1.9)

## 4 Discussion

Two‐dimensional response maps of the silicon detectors Diode E type 60017; the microSilicon type 60023; and the synthetic diamond detector microDiamond type 60019 were obtained using a highly focused proton microbeam. The determined chip diameter of (1.22 ± 0.05) mm for the Diode E is in good agreement with the results of Butler et al.^11^ which have measured an averaged diameter of 1.20 mm and our previous work in which we measured a diameter of 1.22 mm. In case of the microDiamond detector, Butler et al.^11^ investigated three samples and measured diameters between 2.14 mm and 2.21 mm. A further study from Marinelli et al.^13^ investigated ten samples of the microDiamond and determined diameters between 2.17 mm and 2.20 mm. These results correspond well with the diameter of (2.23 ± 0.10) mm measured in this study with the proton technique. Based on this comparison, it can be concluded that the proton microbeam technique presented here is capable of providing accurate geometric data of the diameter of active regions of solid‐state detectors. In addition, this technique allows the determination of the thickness of the active area as well as the dimension and density of the casting compound on top of the chip. This comprehensive characterization distinguishes this technique from previous methods.

The accurate knowledge of the diameter and thickness of the active volume as well as the thickness and density of the casting material on top of the chip is essential for correct Monte Carlo simulations especially with respect to small photon field dosimetry simulations. Fenwick et al.^14^ described the influences of solid‐state detector geometry and material based on the example of small‐field correction factors. Based on a model of the Diode E 60017 detector, they showed an influence of the diameter and thickness of the active silicon volume as well as an influence of the housing of the silicon chip, that is, the casting material above the chip. The influence of the materials above and under the active volume was also investigated in the work of Looe et al.^3^

The results of the microSilicon show significant differences to the former silicon detector Diode E. The diameter of the active volume (1.50 mm) is larger compared to the predecessor (1.22 mm). With respect to small field dosimetry applications, the larger sensitive area leads to a slight increase in the volume‐averaging effect that causes signal reduction at the center of the field. Furthermore, the modeled casting material dimensions of 0.48 mm thickness and density of 1.16 g/cm^3^ were confirmed based on the measurements as shown in Fig. 2. Compared to the predecessor, the casting compound density is highly reduced. According to Fenwick et al.^14^ and Looe et al.,^3^ a decreased density perturbation effect is to be expected in case of the microSilicon detector. The thickness of the active volume (17.8 µm) is smaller than for the predecessor (27.9 µm). The thickness of the active volume (17.8 µm) is also found to be thinner than that of the predecessor (27.9 µm). Nevertheless, the disturbance due to the thickness of the active volume of silicon diode has been shown by Fenwick et al.^14^ to be small.

Furthermore, a recent study^17^ has demonstrated the significant role of radiation‐induced charge imbalance in the contacts and cable of the microDiamond detector on its dose response in small photon fields. Although the thickness of the active volume plays directly a minor role in dose perturbation effects, this type of field size‐dependent radiation‐induced charge will be collected in addition to the charge released in the detector’s sensitive volume. Therefore, its influence on dose response can be modeled correctly only if the dimensions, including the thickness, of the sensitive volume are well known.

## 5 Conclusions

It has been demonstrated that the active volume of commercial silicon and diamond detectors can be fully characterized three‐dimensionally using the highly focused 10 MeV proton microbeam facility of PTB, which includes the determination of two‐dimensional response maps and the active volume’s thickness. These data obtained for the new microSilicon detector can serve as the basis of future Monte‐Carlo simulations, especially in deriving the corresponding small‐field output correction factors.

## Conflicts of interest

Daniela Poppinga and Rafael Kranzer are employees of PTW Freiburg.
